# *ask*MEDLINE: a free-text, natural language query tool for MEDLINE/PubMed

**DOI:** 10.1186/1472-6947-5-5

**Published:** 2005-03-10

**Authors:** Paul Fontelo, Fang Liu, Michael Ackerman

**Affiliations:** 1 Office of High Performance Computing and Communications, National Library of Medicine, 8600 Rockville Pike, Bethesda, Maryland 20894, USA

## Abstract

**Background:**

Plain language search tools for MEDLINE/PubMed are few. We wanted to develop a search tool that would allow anyone using a free-text, natural language query and without knowing specialized vocabularies that an expert searcher might use, to find relevant citations in MEDLINE/PubMed. This tool would translate a question into an efficient search.

**Results:**

The accuracy and relevance of retrieved citations were compared to references cited in BMJ POEMs and CATs (critically appraised topics) questions from the University of Michigan Department of Pediatrics. askMEDLINE correctly matched the cited references 75.8% in POEMs and 89.2 % in CATs questions on first pass. When articles that were deemed to be relevant to the clinical questions were included, the overall efficiency in retrieving journal articles was 96.8% (POEMs) and 96.3% (CATs.)

**Conclusion:**

*ask*MEDLINE might be a useful search tool for clinicians, researchers, and other information seekers interested in finding current evidence in MEDLINE/PubMed. The text-only format could be convenient for users with wireless handheld devices and those with low-bandwidth connections in remote locations.

## Background

*ask*MEDLINE  evolved from the PICO [1] (Patient, Intervention, Comparison, Outcome) search interface, a method of searching MEDLINE/PubMed that encourages the creation of a well-formulated search. [2] Starting from a clinical situation, a clinician is guided through the search process by thinking along PICO elements.

PICO search was developed with the busy clinician in mind, interested in practising evidence-based medicine, but unfamiliar with controlled vocabularies that could make the search more efficient. In an attempt to automate the entry of search terms into PICO elements from a clinical question, we discovered that the user could simply enter a clinical question, then let the search engine retrieve relevant journal articles. The step to allow the user to inspect the correctness of PICO elements was omitted, however, a link is provided to the PICO interface instead, so the user can manually enter search terms if the search results are deemed unsatisfactory.

Although PICO helps in finding recent evidence from MEDLINE/PubMed, some users might still find searching for answers to clinical questions challenging. For some, it might be because it is time consuming to incorporate into their busy practice, difficult to learn, or perhaps, they may feel that it is not clinical enough and may not answer their question. They may find it more convenient to go directly to other subscription-only resources. Although easier to search and filtered for quality, these resources are not without disadvantages. They cover fewer journals than MEDLINE (Medical Literature Analysis and Retrieval System Online), and there is an even greater time lag between the publication of an article and its appearance in these databases than there is with MEDLINE. Other clinicians may just access resources that evidence-based practitioners might consider less evidence-based, (i.e. lesser validity) such as consulting a colleague, or reading a textbook, or perhaps, they may forego searching altogether.

*ask*MEDLINE is intended for the clinician, researcher, or the general public who want to simply ask a question and to skip the challenge of learning how to format it in manner that will make the searching MEDLINE/PubMed efficient. It is a tool that allows the user to search MEDLINE/PubMed using free-text, natural language query, just like one would in a clinical setting, or in a conversation. A user enters a clinical question on a Web browser, and then lets the tool retrieve relevant articles in MEDLINE/PubMed. Links are provided to journal abstracts, full-text articles and related items. Moreover, *ask*MEDLINE is formatted for easy viewing on a wireless handheld device so it can be used while mobile, but will work equally well on a desktop computer.

We report our experience in developing *ask*MEDLINE, and an evaluation study on its potential to retrieve references, using published, evidence-based resources.

## Implementation

*ask*MEDLINE uses a multi-round search strategy. In the first round, the parser ignores punctuation marks and deletes words found on a "stop-word" list. The stop-word list includes PubMed stop words, and other words that we found by experience, to be detrimental to the search. The parser, a PHP script, then sends the modified query to PubMed Entrez' E-Utilities. The Extensible Markup Language (XML) file returned by E-Utilities indicates the category of each term in the query. Terms marked as "All Fields" denote that they are neither Medical Subject Headings (MeSH) terms nor MeSH Subheadings. These terms are checked to determine if they are found in a "MeSH Backup vocabulary." The backup vocabulary includes words other than MeSH terms, such as MeSH descriptors, that are classified as "other eligible entries". If an "All Fields" word is in the backup vocabulary, it remains in the query; if it is not, it is deleted. The remaining terms are sent back to PubMed, again through E-Utilities. Human and English language limits are always applied. If the journal retrieval count after the first round is between 1 and 50,000, the first 20 results are displayed in the user's browser and the search process terminates. Further searches are dependent on the user.

The search may proceed to Round 2 under two conditions: 1) If no journals are found in the first round, a result that could signify that the search was too narrow (i.e., too many terms are searched, too many filters), the "All Fields" words are deleted from the query, even though they are found in the backup vocabulary. Only MeSH Terms and Subheadings remain (Round 2A.) 2) If the first round retrieval count is larger than 50,000 articles (an indication that the search was too broad) the "All Fields" words removed during the first round (words not found in the backup vocabulary) are put back into the query (Round 2B.) Round 2B searches contain all the MeSH terms (or MeSH Subheadings) and "All Fields" words in the original question. The updated query from either 2A or 2B is once again sent to Entrez E-Utilities. Retrieved journal articles are sent to the user.

Similarly, if the count returned from second round is in the range of 1 to 50000, the search process terminates. If the second round count is still equal to 0 (denoting that the search is still too narrow) another list of "No-Go Terms", terms that when removed could result in a successful search is checked. Common MeSH abbreviations, acronyms and words like, "method," "affect," and "lead" are examples of terms on the list. New terms are continuously added to this list as they are encountered. The third round modified query is once again sent to E-Utilities and the retrieved journal articles are sent to the user. A result of 1 to 50000 citations terminates the process and displays the first 20 articles.

If *ask*MEDLINE retrieves only one to four journal articles, a search is automatically done for related articles of the top two articles. All the articles (one to four previous) and the first 25 related articles of the first two are retrieved. As in any of the previous steps, the first 20 are displayed in the browser. In all the search retrieval pages, a link is provided for the user to manually intervene and modify the search process through the PICO interface. Links to related articles, full-text articles and abstracts are shown.

Since November 2002, the British Medical Journal (BMJ) has published a POEM (Patient-Oriented Evidence that Matters) in every issue. [3] POEMs are provided to BMJ by InfoRetriever *ask*MEDLINE was evaluated by comparing its accuracy to retrieve an article cited as a reference in a POEM ("gold standard".) [3] Every POEM has a question with a cited reference that is relevant to the question. We entered every POEM question into *ask*MEDLINE, and for comparison, in Entrez, the integrated, text-based search and retrieval tool for PubMed. New critically appraised topics (CATs) from the University of Michigan, Department of Pediatrics Evidence-Based Pediatrics Web site were also used. [4] Unlike BMJ POEMs, some questions in CATs had more than one cited reference.

The initial search result was examined to determine if the reference cited in a POEM or CAT was among those retrieved. Subsequent steps were taken if the reference article cited was not: 1) If the initial search retrieved journal citations, but not the specific journals cited in a POEM or CAT, the titles and abstracts were scanned to find out if they were relevant (deemed to answer the question.) If they were, related articles were retrieved, and again evaluated to determine if they matched the cited reference. 2) If no journal articles were retrieved, the question was rephrased, then searched again. Retrievals were again examined for the cited articles and relevancy to the clinical question. Overall efficiency was determined by the accuracy in retrieving a cited article and relevance of citations retrieved for citations that did not match cited references.

## Results

### Development of the search tool

**Figure 1:**
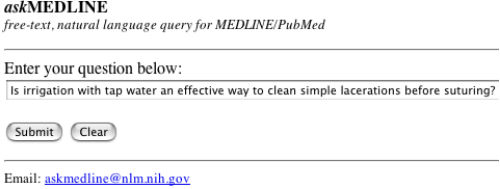
*ask*MEDLINE is formatted for handheld devices, but will work just as well for desktop computers. The search form and retrieval pages are in text only.

A simple, handheld-friendly search interface was created where users can enter free-text, natural language searches (Figure 1.) The results are also provided in text-only mode, optimised for use by the mobile health care personnel using handheld devices. Links to abstracts, related citations in MEDLINE and full-text articles are provided. Full-text links to journal publishers are not text-based, and may require journal subscription or fees for viewing.

### Evaluation of search retrievals

Clinical questions in 95 POEMs and 28 CATs were searched. After first pass, *ask*MEDLINE found 62% of the cited articles in POEMs, while Entrez retrieved close to 14% (Table 1.) When related articles were searched, 11.6% more were found by *ask*MEDLINE (8.4% in Entrez.) When three questions were rephrased, askMEDLINE, but none in Entrez retrieved two of the specific cited references, although relevant references were found to one of the questions. For 20 questions, *ask*MEDLINE did not find the specific cited reference, but it found journal citations that were deemed relevant and would be useful in answering the question. Entrez obtained citations for 16 (16.8%) questions that were considered relevant.

**Table 1:** POEMs Evaluation Study. A comparison of the accuracy and efficiency of *ask*MEDLINE and Entrez PubMed in retrieving an exact match to cited references in POEMs in BMJ.

**Search Step**	***ask*MEDLINE exact match retrieved/total questions (% total)**	**Entrez PubMed exact match retrieved/total questions (% total)**
A. Match at first pass	59/95 (62.1)	13/95 (13.7)
B. Match after a related citation search	11/95 (11.6)	8/95 (8.4)
C. Match after question rephrase	2/95 (2.1)	0/95 (0)
**Total exact match after A, B, and C**	**75.8%**	**22.1**
No match after A, B, and C, but relevant articles retrieved	20/95 (21)	16/95 (16.8)
**Overall efficiency**	**96.8%**	**38.9%**
Citations retrieved, but not matched or relevant	3/95 (3.1)	6/95 (6.3)
No citations retrieved	0/95 (0)	52/95 (54.7)
**Overall retrieval failure**	**3.1%**	**61%**

Overall, *ask*MEDLINE retrieved 72/95 exact matches of cited references (gold standard) in POEMs, an accuracy of 75.8%, while Entrez' accuracy was 22% (21/95.) If citations that are not the same as those cited in POEMs, but are relevant and considered satisfactory for answering the clinical question are included, *ask*MEDLINE's total efficiency is 96.8%. Entrez' total efficiency for finding specific and relevant citations for BMJ POEMs is 38.9% (21 specific and 16 relevant citations found.)

Although citations were retrieved for all POEM questions by *ask*MEDLINE, three searches did not find exact matches or relevant articles (3.1%), while Entrez' results were not relevant (6.3%) for six questions. No citations were found for 52 (54% of total) questions by Entrez.

University of Michigan's CATs yielded a similar total efficiency as POEMs, 96.3%, while it was 14.3% for Entrez (Table 2.) First pass retrieval was 64.2% (Entrez 3.6%) and citation retrievals for related citations was 10.7% (Entrez 3.6%.) Four of six questions rephrased added 14.3% to the total efficiency of *ask*MEDLINE, while it added 7.1% to Entrez. Almost 7% of the searches retrieved relevant citations to rephrased or related articles, but none in Entrez. For CATs' questions, *ask*MEDLINE found 89.2% of cited references, but 14.3% for Entrez. In 21/28 questions, Entrez did not provide a specific or relevant citation, but it was only for one question with *ask*MEDLINE.

**Table 2:** CATs Evaluation Study. The accuracy and efficiency of *ask*MEDLINE and Entrez in retrieving an exact match to cited references in CATs' questions from the University of Michigan, Department of Pediatrics Evidence-Based Pediatrics Web site.

**Search Step**	***ask*MEDLINE exact match retrieved/total questions (% total)**	**Entrez PubMed exact match retrieved/total questions (% total)**
A. Match at first pass	18/28 (64.2)	1/28 (3.6)
B. Match after a related citation search	3/28 (10.7)	1/28 (3.6)
C. Match after question rephrase	4/28 (14.3)	2/28 (7.1)
**Total exact match after A, B, and C**	**89.2%**	**14.3%**
No match after A, B, and C, but relevant articles retrieved	2/28 (7.1)	0/28 (0)
**Overall efficiency**	**96.3%**	**14.3%**
Citations retrieved, but not matched or relevant	1/28 (3.6)	3/28 (10.7)
No citations retrieved	0/28 (0)	21/28 (75)
**Overall retrieval failure**	**3.6%**	**85.7%**

## Discussion

*ask*MEDLINE is part of a project to develop easy-to-use resources at the point of care that has the functionality of an expert searcher. [5] Special consideration was given to healthcare personnel who use handheld devices in wireless environments, hence the emphasis on text rather than images. The results of the evaluation using POEMs in BMJ and CATs from the University of Michigan seem to indicate that it may a useful addition.

MEDLINE now contains more than 13 million citations from over 4,000 journals, with approximately 40,000 added monthly. The Internet also holds millions of Web pages that archive medically related information. The task of the user then is to find the information one needs, sift through the good, bad and dangerous, and after considering other factors (patient information, laboratory tests, personal experience) apply them to the management of a patient. This is the practice of evidence-based medicine. Providing decision support for evidence-based practice at the point of care is the goal of *ask*MEDLINE.

Several special vocabularies were considered and tested for *ask*MEDLINE- - Unified Medical Language System (UMLS), International Classification of Diseases: 9th revision (ICD9), but MeSH was selected because it is the controlled vocabulary used for indexing articles for MEDLINE/PubMed. MeSH terminology presents a reliable means of retrieving information that may use different terminologies for the same concept. The multi-round algorithm was developed through testing.

The difference in accuracy rates between the POEMs (75.8%) and CATs (89.2%) is most likely explained by the greater number of cited references in CATs. POEMs only had one, but CATs had one or more per question. We considered a single match to a reference is a positive count. The overall efficiency of finding a citation match in POEMs and CATs was consistent, around 96%. We are unable to account for the discordance with between POEMs (38.9%) and CATs (14.3%) questions.

Some POEMs' questions did not return exact matches although retrievals may have been relevant, but when the questions were reformulated, positive matches were obtained. One example was the questions, "Is low dose aspirin safe and effective for the prevention of thrombotic complications in patients with polycythaemia vera?" which returned 51 relevant citations but no exact match. When it was rephrased to, "Does low dose aspirin prevent thrombotic complications in polycythaemia vera?" the second citation matched the cited POEMs reference. The question, "Is a prolonged period of antithrombotic pretreatment effective for reducing adverse outcomes in patients with unstable coronary syndromes?" returned close to 40,000 citations, but when rephrased to, "Is prolonged antithrombotic treatment indicated in unstable coronary syndromes before intervention?" the first citation was an exact match.

There were questions that did not match despite attempts to rephrase them. One, "Is a one day treatment of Helicobacter pylori as effective as a seven day regimen in patients with dyspepsia?" The citations retrieved were all relevant, but none was the exact match because there were many treatment regimens variations. Some questions were quite easily modified to obtain exact matches, like, "Does azithromycin given to patients with acute coronary syndromes prevent recurrent ischaemia?". But by simply deleting the "s" in syndromes, the first of 25 relevant citations was a match. Other examples are shown in Table 3.

**Table 3:** Examples of rephrased questions. Minor modifications of some questions increased retrievals of exact matches of cited references. ^a^POEMs, ^b^CATs

Original question	Result before rephrasing question (Citation match/total retrieved)	Rephrased question	Result after rephrasing question (Citation match/total retrieved)
^a^What is the risk that any given mole will become a melanoma?	0/31	What is the risk of any given mole transforming into a melanoma?	1/50
^a^Are ear temperatures reliable?	0/511	Is measurement of ear temperature reliable?	6/23
^b^In children with an acute febrile illness, what is the efficacy of single-medication therapy with acetaminophen or ibuprofen compared with combination therapy combining the two medications in reducing fever while avoiding adverse effects?	0/0	In children with fever, is acetaminophen or ibuprofen alone better than combined, while avoiding adverse effects?	8/25

Currently, there are not many free-text, natural query search engines that are specifically developed for MEDLINE/PubMed. Requiring clinicians to learn and master search methods for efficient searching is unrealistic. [6] It is even more important for the general public, unfamiliar with medical terminology, who are seeking medical information from MEDLINE/PubMed in increasing numbers, but who frequently do not use effective strategies. [7] Providing a natural language search tool that mimics a clinician's practice habits at the point of care, but with the functionality of an expert medical librarian might be beneficial. *ask*MEDLINE also uses MeSH and omits variation that other search engines have that may impact search results as some have found. [8]

Google is a very powerful search engine that searches the entire World Wide Web, including biomedical Web sites. There are ongoing discussions in biomedical lists on the merits of Google and PubMed. They search different databases and comparisons between the two are not valid. *ask*MEDLINE is a more modest tool and certainly not comparable to either of the two.

## Conclusion

Evaluation of *ask*MEDLINE based on two collections of evidence-based resources suggests that it may be a useful resource for clinicians, researchers and the general users interested in finding relevant medical information. Its text-only format will make it convenient for users of wireless handheld devices to use it in the clinical setting where wireless Internet access is available. It will also be helpful for those in with slow connections to the Internet especially those in remote locations in developing countries. Efforts to improve its functionality and clinical usefulness for evidence-based medicine are continuing.

## Availability and requirements

**Project name: **Development of evidence-based medicine tools

**Project home page: **

**Operating system: **platform independent

**Programming language: **PHP

**Other requirements: **Apache, MySQL, PHP

**License: **Free, anyone may use the service

**Any restrictions to use by non-academics: **None, anyone may use the service

## Competing interests

The author(s) declare that they have no competing interests

## Authors' contributions

PF conceived of the study and design, participated in the development of the search tool, carried out the evaluation studies and drafted the manuscript. FL participated in the design of the study and wrote the search script. MA participated in the study design, evaluation study and gave final approval of the version to be published. All authors read and approved the final manuscript.

## Pre-publication history

The pre-publication history for this paper can be accessed here:


